# A Pilot Study for “In Vitro” Testing the Surface Conditioning Effects on CAD/CAM Hybrid Nanoceramic Adhesion

**DOI:** 10.3390/dj14010036

**Published:** 2026-01-06

**Authors:** Georgi Veselinov Iliev, Lucian Toma Ciocan, Vlad Gabriel Vasilescu, Gaudențiu Vărzaru, Florin Miculescu, Ana Maria Cristina Țâncu, Marina Imre, Silviu Mirel Pițuru

**Affiliations:** 1 Department of Prosthodontics, Faculty of Dentistry, Medical University of Sofia, 1 Georgi Sofiyski Street, 1431 Sofia, Bulgaria; ilievdent@gmail.com; 2 Department of Dental Prostheses Technology, Faculty of Dentistry, “Carol Davila” University of Medicine and Pharmacy, 37 Dionisie Lupu Street, District 2, 020021 Bucharest, Romania; 3 Center of Technological Electronics and Interconnection Techniques (CETTI), Faculty of Electronics, Telecommunications and Information Technology, National University of Science and Technology Politehnica Bucharest, 011061 Bucharest, Romania; gaudentiu.varzaru@cetti.ro; 4 Faculty of Material Science and Engineering, National University of Science and Technology Politehnica Bucharest, 011061 Bucharest, Romania; f_miculescu@yahoo.com; 5 Department of Prosthodontics, Faculty of Dentistry, “Carol Davila” University of Medicine and Pharmacy, 37 Dionisie Lupu Street, District 2, 020021 Bucharest, Romania; anamaria.tancu@umfcd.ro (A.M.C.Ț.); marina.imre@umfcd.ro (M.I.); 6 Discipline of Organization, Professional Legislation and Dental Office Management, Faculty of Dentistry, “Carol Davila” University of Medicine and Pharmacy, 37 Dionisie Lupu Street, District 2, 020021 Bucharest, Romania; silviu.pituru@umfcd.ro

**Keywords:** surface treatments, hybrid nano-ceramics, adhesion

## Abstract

**Background/Objectives**: The clinical application of CAD/CAM restorative materials continues to evolve due to increasing demand for aesthetic, durable, and minimally invasive indirect restorations. Hybrid nanoceramics, such as Grandio disc (VOCO GmbH, Cuxhaven, Germany), are increasingly used in indirect restorative dentistry due to their favourable combination of mechanical strength, polishability, wear resistance, and bonding potential. One challenge associated with adhesive protocols for CAD/CAM materials lies in achieving durable bonds with resin cements. Extensive post-polymerization during fabrication reduces the number of unreacted monomers available for chemical interaction, thereby limiting the effectiveness of traditional adhesive strategies and necessitating specific surface conditioning approaches. This study aimed to evaluate, in a preliminary, non-inferential manner, the influence of several combined conditioning protocols on surface micromorphology, elemental composition, and descriptive SBS trends of a CAD/CAM hybrid nanoceramic. This work was designed as a preliminary pilot feasibility study. Due to the limited number of specimens (two discs per protocol, each providing two independent enamel bonding measurements), all bond strength outcomes were interpreted descriptively, without inferential statistical testing. This in vitro study investigated the effects of various surface conditioning protocols on the adhesive performance of CAD/CAM hybrid nanoceramics (Grandio disc, VOCO GmbH, Cuxhaven, Germany) to dental enamel. Hydrofluoric acid (HF) etching was performed to improve adhesion to indirect resin-based materials using two commercially available gels: 9.5% Porcelain Etchant (Bisco, Inc., Schaumburg, IL, USA) and 4.5% IPS Ceramic Etching Gel (Ivoclar Vivadent, Schaan, Liechtenstein), in combination with airborne-particle abrasion (APA), silanization, and universal adhesive application. HF may selectively dissolve the inorganic phase, while APA increases surface texture and micromechanical retention. However, existing literature reports inconsistent results regarding the optimal conditioning method for hybrid composites and nanoceramics, and the relationship between micromorphology, elemental surface changes, and adhesion remains insufficiently clarified. **Methods**: A total of ten composite specimens were subjected to five conditioning protocols combining airborne-particle abrasion with varying hydrofluoric acid (HF) concentrations and etching times. Bonding was performed using a dual-cure resin cement (BiFix QM) and evaluated by shear bond strength (SBS) testing. Surface morphology was examined through environmental scanning electron microscopy (ESEM), and elemental composition was analyzed via energy-dispersive X-ray spectroscopy (EDS). **Results**: indicated that dual treatment with HF and sandblasting showed descriptively higher SBS, with values ranging from 5.01 to 6.14 MPa, compared to 1.85 MPa in the sandblasting-only group. ESEM revealed that higher HF concentrations (10%) created more porous and irregular surfaces, while EDS indicated an increased fluorine presence trend and silicon reduction, indicating deeper chemical activation. However, extending HF exposure beyond 20 s did not further improve bonding, suggesting the importance of protocol optimization. **Conclusions**: The preliminary observations suggest a synergistic effect of mechanical and chemical conditioning on hybrid ceramic adhesion, but values should be interpreted qualitatively due to the pilot nature of the study. Manufacturer-recommended air abrasion alone may provide limited adhesion under high-stress conditions, although this requires confirmation in studies with larger sample sizes and ageing simulations. Future studies should address long-term durability and extend the comparison to other hybrid CAD/CAM materials and to other etching protocols.

## 1. Introduction

The clinical applications of CAD/CAM restorative materials have evolved due to rising patient demands for aesthetic, durable, and minimally invasive indirect restorations, as well as improvements in adhesive protocols. Owing to their combination of mechanical strength and aesthetic potential, hybrid nanoceramics are increasingly viewed as viable alternatives to traditional indirect restorative materials [1,2]. The demand for hybrid nanoceramics and other resin-based CAD/CAM materials continues to rise and is increasingly favoured due to their enhanced polishability, wear resistance, and improved bonding performance [3].

One of the main limitations of adhesive protocols involving CAD/CAM materials is the difficulty in achieving durable and stable bonds with luting resin cements [4,5]. Due to the extensive post-curing involved in CAD/CAM fabrication, these materials exhibit a high degree of polymerization, leaving few unreacted monomers available for chemical adhesion. The high degree of polymerization in CAD/CAM materials reduces their susceptibility to traditional adhesive strategies and requires specific surface treatments to enhance micromechanical interlocking or chemical interactions [6].

Hydrofluoric acid (HF) etching, airborne-particle abrasion (APA), silanization, and the application of universal adhesives, including functional monomers such as MDP (10-methacryloyloxydecyl dihydrogen phosphate), have been indicated as methods to improve adhesion to indirect resin composites [7,8]. By selectively dissolving the glass phase in hybrid composites or ceramics, hydrofluoric acid generates a roughened surface that improves mechanical retention [9].

Air abrasion, using 30–50 µm Al_2_O_3_ particles, is commonly used to create surface imperfections and remove impurities [10].

Though their efficiency may vary depending on the amount of silica in the substrate and the specific application technique, silane coupling agents can facilitate bonding between the inorganic filler of CAD/CAM materials and resin cements [11,12].

In recent years, manufacturers have introduced a variety of universal adhesives and silane-containing agents that aim to simplify clinical protocols and improve the predictability of bond strength outcomes. However, studies have reported inconsistent results regarding the durability and magnitude of adhesion when combining different surface treatments with various adhesive systems [10,11,13]. Recent systematic evaluations confirmed that resin-matrix and hybrid CAD/CAM ceramics exhibit substantial variability in bonding outcomes depending on the adhesive system and surface conditioning protocol applied [14,15]. Moreover, most in vitro studies evaluate only immediate bond strength without adequately addressing the micromorphological and compositional changes induced by each protocol, which are crucial for understanding the adhesive mechanism at the interface [16].

Therefore, comprehensive evaluations combining mechanical testing and surface characterization techniques such as scanning electron microscopy (SEM) and energy-dispersive X-ray spectroscopy (EDS) are needed to provide insight into the morphological changes and chemical compositions that influence adhesion [17,18]. Given the exploratory pilot nature of the present work, the outcomes were intended to provide preliminary, qualitative adhesion tendencies rather than statistically generalizable conclusions.

Considering the increasing clinical demand for CAD/CAM hybrid nanoceramic restorations, optimizing bonding protocols has become essential to ensure long-term performance, especially since current literature and manufacturer guidelines lack consensus regarding the most effective surface conditioning techniques. A recent scoping review further emphasized the absence of a universally accepted conditioning strategy for resin-matrix CAD/CAM hybrid nanoceramics [15]. Therefore, this study aimed to evaluate how combined mechanical and chemical conditioning protocols influence the micromorphology and adhesive behaviour of hybrid nanoceramic materials used in indirect restorations.

Although numerous studies examine surface treatments for indirect CAD/CAM polymer-based materials, most focus on high-viscosity composites or polymer-infiltrated ceramic networks (PICNs), and there is a lack of integrated chemical–mechanical analyses linking micromorphology, elemental alterations, and bonding efficacy. Hybrid nanoceramics with increased filler content, such as Grandio discs, exhibit unique physicochemical properties when exposed to hydrofluoric acid (HF); yet existing research provides inconsistent guidance on the ideal HF concentration and etching period. These inconsistencies are supported by recent findings showing that HF concentration and exposure time influence etching depth and surface integrity in resin-matrix ceramics [19]. Furthermore, most research evaluates bonding strength in isolation, failing to connect micromechanical alterations to elemental surface reactivity. A comprehensive assessment of ESEM/EDS and shear bond performance is essential to clarify the impact of HF parameters on chemical activation and interfacial adhesion.

### Purpose of the Study

Based on these considerations, the current study aimed to evaluate, in vitro, the effect of various surface conditioning protocols—combining mechanical and chemical treatments—on the adhesive performance of CAD/CAM hybrid nanoceramics, with particular focus on their micromorphological changes and bond strength to hard dental tissues.

## 2. Materials and Methods

### 2.1. Analyzed Materials

Ten circular samples (6 mm diameter, 2 mm thickness) were obtained by milling a nanohybrid CAD/CAM discs (Grandio disc, VOCO GmbH, Cuxhaven, Germany; shade A2) using a Planmeca PlanMill 50S milling unit (Planmeca Oy, Helsinki, Finland), under constant water cooling. The luting system consisted of:

- Futurabond U (VOCO GmbH, Cuxhaven, Germany)—applied on enamel surfaces;
- Ceramic Bond (VOCO GmbH, Cuxhaven, Germany)—applied on composite samples;
- BiFix QM (VOCO GmbH, Cuxhaven, Germany)—dual-cure resin cement.
- Hydrofluoric acid (HF). Two concentrations were used for surface conditioning: 4.5% HF IPS ceramic etching gel (manufacturer: Ivoclar Vivadent, Schaan, Liechtenstein, lot: Z03D6W) and 9.5% Porcelain Etchant (manufacturer: Bisco, Inc., 1100 W. Irving Park Rd. Schaumburg, IL, USA, lot: 2400014784). Etching was performed in a certified chemical fume hood with full PPE (HF-rated gloves over nitrile, face shield, lab coat). After etching, specimens were immediately rinsed under distilled water for 30 s and then immersed in an ultrasonic bath with distilled water for 60 s to aid the removal of soluble reaction products. All HF waste was collected and neutralized in accordance with institutional SOPs before disposal.

### 2.2. Tooth Sample Preparation

This investigation was designed as a pilot feasibility study, with the explicit purpose of exploring whether measurable micromorphological and elemental changes produced by different conditioning protocols could be correlated with preliminary trends in adhesive behaviour. The goal was not to establish statistical differences or clinically validated SBS values. Each conditioning protocol was applied to two CAD/CAM discs, and each disc provided two independent bonded enamel surfaces (buccal and lingual). These replicates were treated as independent measurements exclusively to expand preliminary feasibility data, without inferential statistical interpretation.

Due to material availability and equipment constraints, each conditioning protocol was applied to two milled composite discs, and each disc provided two independent bonded enamel surfaces (buccal and lingual). These two surfaces were treated purely as independent replicates, not as anatomically distinct groups, yielding four descriptive SBS measurements per protocol. This approach was selected to: maximize the number of surface measurements per disc; minimize variability related to disc-to-disc structural heterogeneity; generate preliminary data for feasibility assessment before committing resources to a full statistical study. Because the objective was exploratory, no sample size calculation was performed, and no inferential statistics were applied. All SBS values are reported descriptively.

This survey was approved by the Scientific Research Ethics Committee of “Carol Davila” University of Medicine and Pharmacy, Bucharest, Romania (Protocol number: 30317; date: 24 October 2025). The scientific study was conducted in accordance with the Declaration of Helsinki of 1975, revised in 2013.

Five human 3rd molars with the indication to be extracted (for orthodontic reasons) were used as the opposing bonding substrate. Teeth were selected to be free of carious lesions, enamel defects, or visible cracks. After mechanical cleaning, teeth were stored for two weeks in 0.5% chloramine-T, then immersed in distilled water (DIN ISO 3696, grade 3) at 4 °C and used within four weeks post-extraction.

This study was designed as a preliminary pilot study, with two discs per conditioning protocol, each tested on two independent enamel surfaces (buccal and lingual), yielding four valid SBS measurements per group. The buccal and lingual enamel surfaces were flattened using a diamond disc under water cooling. Smear layer removal was standardized by polishing with 600-grit silicon carbide abrasive paper. Surfaces were then rinsed with distilled water and air-dried.

The limited number of specimens per group (*n*2 discs generating four bonding surfaces) was intentionally selected because this investigation was designed as a preliminary proof-of-concept aiming to correlate elemental and micromorphological changes qualitatively (EDS/ESEM) with adhesive trends. No sample size calculation was performed, as the objective of the present work was not to draw statistical inferences but to identify conditioning parameters with potential clinical relevance for future large-scale experiments. Accordingly, all SBS values are reported descriptively, without assumptions of statistical representativeness.

### 2.3. Surface Treatment Protocol for Composite Discs

Samples were randomly assigned to five experimental groups, depending on the surface conditioning method (Table 1).

The limited number of specimens per group was intentionally selected for a preliminary proof-of-concept evaluation focused on qualitative surface characterization (ESEM/EDS). Therefore, the results are interpreted descriptively, without statistical inference, to illustrate the main trends produced by each conditioning protocol.

HF (4.5% IPS ceramic etching gel Ivoclar Vivadent, Schaan, Liechtenstein and 9.5% Porcelain Etchant Bisco). was applied using microbrushes in a ventilated fume hood. After the designated time (20 s or 60 s), the surface was thoroughly irrigated with distilled water for 30 s, followed by 60 s ultrasonic bathing in distilled water, air-drying, and immediate silanization. These steps were chosen to minimize any removable fluoride-containing residues on the surface.

### 2.4. Cementation Procedure

**Figure 1:**
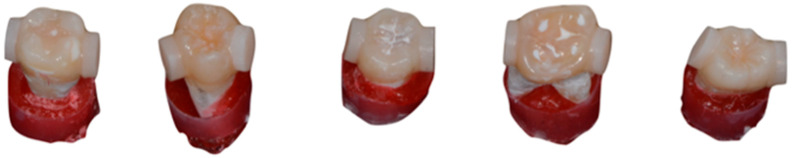
Extracted human molars with CAD/CAM composite discs cemented to buccal and lingual enamel surfaces.

Futurabond U was applied on etched enamel surfaces (BISCO’s Etch-37 w/BAC is a 37% semi-gel phosphoric acid etchant available with Benzalkonium Chloride (BAC), Bisco, Inc., 1100 W. Irving Park Rd. Schaumburg, IL, USA) according to the manufacturer’s instructions, followed by light-curing for 20 s. BiFix QM dual-cure resin cement was applied to the composite discs, which were then pressed against the tooth surface. Excess cement was carefully removed with a microbrush. Final polymerization was carried out for 40 s using an LED curing unit (Bluephase G2, Ivoclar Vivadent, Schaan, Liechtenstein; 1200 mW/cm^2^). A representative image showing the five molars with cemented composite discs is presented in Figure 1.

### 2.5. Storage and Shear Bond Strength Testing

**Figure 2:**
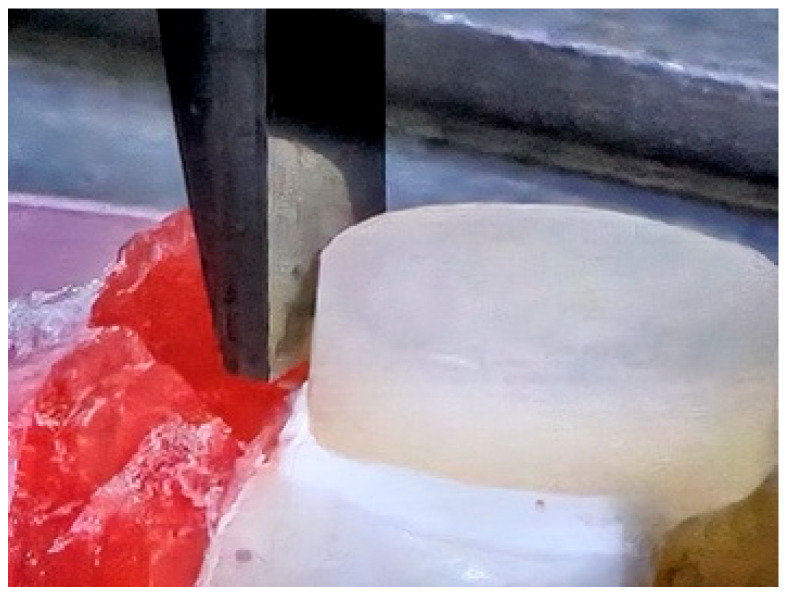
Shear bond strength testing setup using the Condor 70 multifunctional tester with a notched-edge loading spatula.

The cemented assemblies were stored in distilled water at room temperature for 24 h. Shear bond strength (SBS) testing was performed using a Condor 70 multifunctional bond tester (XYZTEC, Panningen, The Netherlands) multifunctional bond tester, in accordance with DIN 13990-2/2009. Figure 2 illustrates the positioning of the loading spatula during force application.

The loading spatula was positioned parallel to the adhesive interface, applying force at a crosshead speed of 1 mm/min until failure occurred. The applied load (in N) was divided by the bonded surface area (28.27 mm^2^) to calculate SBS in MPa. Both buccal and lingual sides of each specimen were tested independently.

### 2.6. SEM and EDS Analysis

Representative specimens from each group were examined using a Philips XL-30 ESEM (Philips, Eindhoven, The Netherlands). Image acquisition and elemental analysis were performed using the manufacturer’s proprietary software provided with the Philips XL-30 ESEM system (Philips, Eindhoven, The Netherlands). Images were acquired under low vacuum, at 30 kV, without additional sample coating. Elemental surface analysis was performed using integrated energy-dispersive X-ray spectroscopy (EDS) to determine the composition and presence of residuals after surface treatment. EDS spectra were acquired from 3 randomly selected areas on each treated surface (*n* = 3 per protocol), using a spot mode of 10–20 μm. Measurements were consistently taken from flat, non-defective regions to avoid topographic artifacts. Elemental analysis was performed in a comparative, semi-quantitative manner, particularly for light elements (C and O), which are known to exhibit limited accuracy when measured by EDS.

## 3. Results

### 3.1. ESEM Analysis

**Figure 3:**
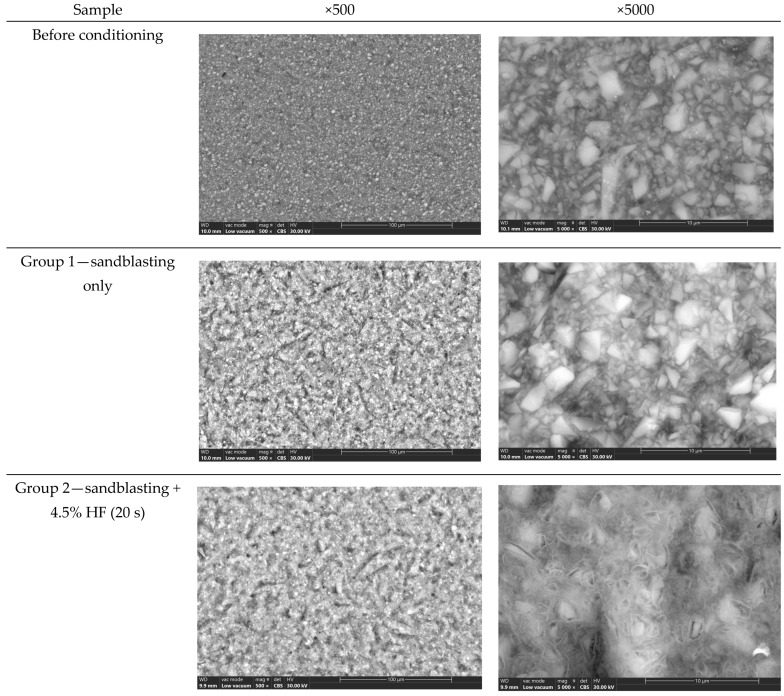

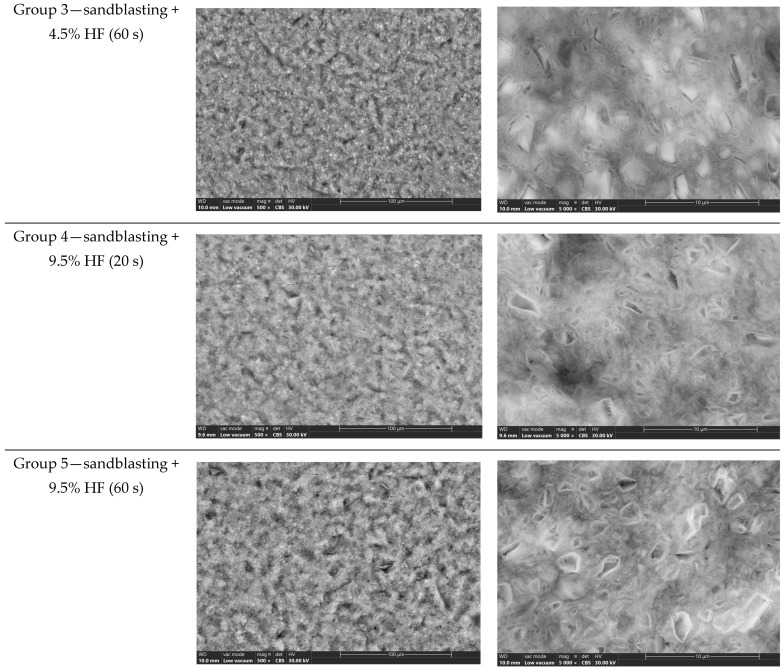
Representative ESEM micrographs (×500, ×5000) of CAD/CAM composite surfaces before and after conditioning.

**Figure 4:**
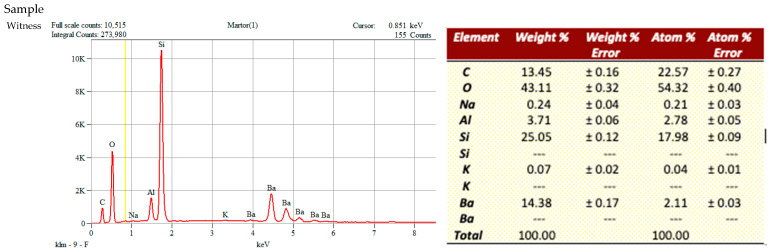

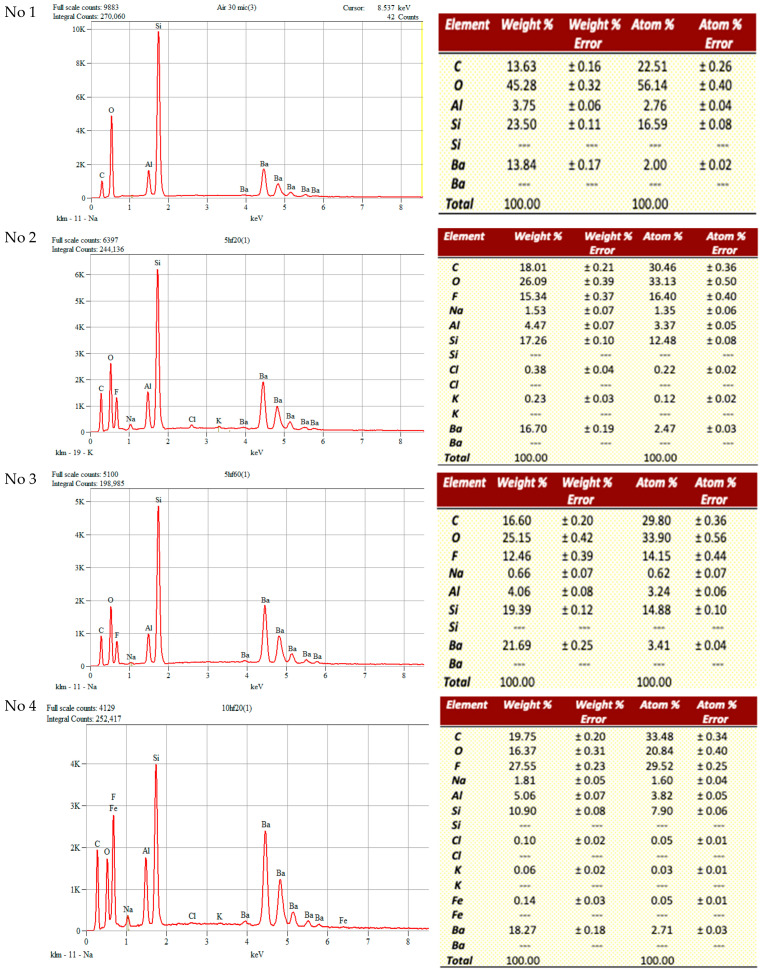

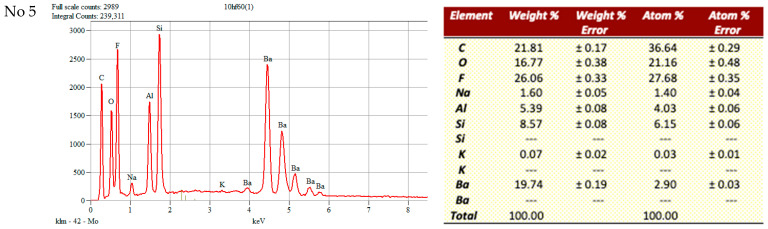
Elemental composition (Weight %) by EDS before bonding for Groups 1–5. Group 1—sandblasting only; Group 2—sandblasting + 4.5% HF (20 s); Group 3—sandblasting + 4.5% HF (60 s); Group 4—sandblasting + 9.5% HF (20 s); Group 5—sandblasting + 9.5% HF (60 s).

Groups 1–5 correspond to the following surface-conditioning protocols (Table 1): Group 1—sandblasting only; Group 2—sandblasting + 4.5% HF (20 s); Group 3—sandblasting + 4.5% HF (60 s); Group 4—sandblasting + 9.5% HF (20 s); Group 5—sandblasting + 9.5% HF (60 s). These references are used consistently throughout Figure 3 and Figure 4.

Prior to the adhesive procedure, the micromorphology of the CAD/CAM composite surfaces was assessed using environmental scanning electron microscopy (ESEM). Figure 3 show typical ESEM images of the five treated groups under different combinations of sandblasting and hydrofluoric acid (HF) application as well as the untreated (witness) group.

The ESEM images showed progressively more pronounced surface roughening as the HF concentration increased. The observed micromorphological changes appeared consistent with selective dissolution of the inorganic phase. EDS revealed relative increases in fluorine content in high-concentration HF groups, although these findings are semi-quantitative.

SBS measurements ranged descriptively from 1.85 to 6.14 MPa across all groups. As expected in a feasibility study with a very small n, the values showed variability. The differences observed between conditioning protocols should be interpreted solely as qualitative signals, not as statistically supported outcomes.

A relatively uniform and homogeneous morphology was observed on the witness surface, with minimal surface irregularities. By contrast, dependent on the HF concentration and etching time, all treated groups showed rougher surfaces.

Group No. 1, which consisted solely of sandblasting, exhibited a surface with a minor texture and uniformly distributed microporosities. With well-defined microcavities and etched resin matrix areas, Groups No. 2 and No. 3, etched with 4.5% HF for 20 and 60 s, respectively, showed an apparent increase in surface roughness.

Groups No. 4 and No. 5, which were subjected to 9.5% HF for comparable durations, exhibited the most pronounced microstructural modifications. The surface structure was highly porous and irregular, indicating that the organic matrix and filler particles were selectively dissolved and that HF penetrated the surface more deeply.

These results demonstrate that surface pretreatment techniques significantly alter the topography of CAD/CAM composite blocks and may improve micromechanical retention prior to adhesive bonding [20].

### 3.2. EDS Analysis Before Bonding

Prior to bonding, the elemental composition of the CAD/CAM composite surfaces was analyzed using Energy-Dispersive X-ray Spectroscopy (EDS) for all five experimental groups and the untreated control (witness). The witness surface presented the typical distribution of carbon (13.45 wt %), silicon (25.00 wt %), and oxygen (43.11 wt %), consistent with the inorganic polymeric matrix of resin nanoceramics [21], along with traces of barium, sodium, and aluminum.

After sandblasting only (Group 1), minimal chemical changes were detected compared with the untreated control, indicating that airborne-particle abrasion alone had little effect on elemental composition.

Groups 2 and 3, treated with 4.5% HF for 20 s and 60 s after sandblasting, showed slight reductions in silicon and oxygen together with limited fluorine uptake, suggesting that low HF concentration produced only superficial reactivity.

In contrast, Groups 4 and 5, treated with 9.5% HF for 20 s and 60 s, exhibited pronounced increases in fluorine content (27.55 wt % in Group 4 and 26.06 wt % in Group 5) accompanied by decreases in silicon and oxygen levels. These changes confirm that higher HF concentration caused deeper dissolution of the silicate phase and greater chemical activation of the surface [22,23].

Despite both groups receiving 9.5% HF treatment, Group 4 (20 s) had significantly higher fluorine content compared to Group 5 (60 s). This observation aligns with the recognized phenomenon of surface over-etching, wherein extended exposure to HF erodes the initially created fluoride-rich layer, which is subsequently eliminated during rinsing, leading to a diminished measurable fluorine concentration despite more profound subsurface modification [22,23,24,25,26,27,28]. A shorter etching duration thus maintains a more stable fluorine-rich surface coating, while prolonged exposure primarily improves etching depth rather than the preservation of surface fluorides [2,7,22,28,29].

Residual elements such as aluminum, potassium, and barium were variably present across samples, reflecting the heterogeneous nature of the hybrid material and localized effects of etching. EDS detects elements, not chemical species; hydrogen is not detected, and fluorine is detected irrespective of its bonding state. Therefore, an F signal indicates surface fluorine presence (e.g., fluoride-containing reaction products) but cannot identify HF molecules. Combined with our extensive aqueous rinsing and ultrasonic cleaning, and given HF’s high volatility/solubility, residual HF on the surface is unlikely; nonetheless, EDS alone cannot prove the chemical speciation of fluorine. Overall, the EDS findings corroborate the ESEM observations (Figure 3), indicates that surface pre-treatment with high-concentration HF may substantially modify the elemental composition, which may enhance both chemical reactivity and micromechanical retention of CAD/CAM hybrid nanoceramics.

These values represent relative elemental trends between conditioning protocols rather than absolute compositional quantification, due to the known limitations of EDS for low-Z elements (C, O).

### 3.3. Shear Bond Strength

The adhesive performance between the CAD/CAM composite discs and the enamel substrate was evaluated using shear bond strength (SBS) testing performed with a Condor 70 multifunctional bond tester. Each specimen was tested on two opposing surfaces (buccal and lingual). A total of ten valid measurements were obtained and included in the analysis. The adhesive testing was intentionally performed on enamel rather than dentin, as the clinical indications of hybrid CAD/CAM nanoceramics such as Grandio disc primarily involve minimally invasive preparations where adhesion is expected to occur predominantly on enamel. Therefore, evaluating adhesion on dentin would not have been clinically relevant within the scope of this study.

Bond forces, initially expressed in kilogram-force (KgF), were converted into megapascals (MPa) by calculating the applied force in newtons and dividing by the bonding surface area (28.27 mm^2^). The full dataset, along with the calculated values, is presented in Table 2.

**Figure 5:**
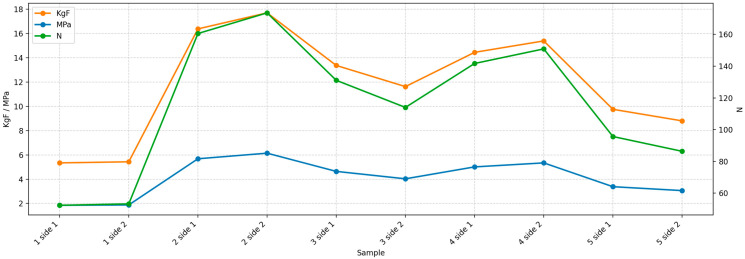
Shear bond strength values (MPa) for individual specimens.

The individual SBS values obtained for each surface tested are illustrated in Figure 5. The SBS values ranged from 1.85 to 6.14 MPa, with a mean value of 4.101 ± 1.55 MPa. This wide distribution reflects variation between samples, potentially influenced by microtopography, cement handling, or curing consistency.

## 4. Discussion

The present pilot feasibility study aimed to qualitatively assess how combined mechanical and chemical conditioning affects the surface characteristics and preliminary adhesive behaviour of a hybrid CAD/CAM nanoceramic. Although the number of samples per group was intentionally limited to obtain descriptive trends, several observations align with existing literature and provide relevant context for future investigations. The current research observations indicated descriptively that shear bond strength values were significantly higher when hydrofluoric acid etching was used with sandblasting than sandblasting alone. These results are consistent with prior studies [25,26] demonstrating that etching increases selective dissolution of the ceramic phase, thereby improving micromechanical linking, increasing surface roughness enabling stronger micromechanical interlocking and strengthening bonding durability.

The improved micromechanical retention observed following combined therapies supports the theory that manufacturer-suggested procedures should be customized based on clinical circumstances, especially in cases of projected high occlusal forces.

Technical data sheets provided by CAD/CAM ceramic manufacturers could indicate a simple approach to surface conditioning, relying solely on air abrasion. This approach disregards clinical factors, including occlusal stress, bonding substrate, and restorative thickness, though. Including an etching step with hydrofluoric acid in our approach not only changed the surface morphology—as demonstrated in ESEM images—but additionally increased fluorine absorption, implying deeper surface reactivity (verified using EDS).

The ESEM analysis revealed that the application of HF—particularly at 9.5% concentration—produced marked increases in surface roughness, porosity, and selective dissolution of both organic and inorganic phases. This observation is consistent with previous reports indicating that HF can effectively alter the superficial structure of hybrid ceramics and resin-based CAD/CAM materials by dissolving silica-rich components and exposing filler clusters [25,27,28]. Similar HF-induced microstructural degradation and filler exposure patterns have also been demonstrated in recent analyses of aged hybrid ceramics and CAD/CAM nanoceramics subjected to mechanical–chemical pretreatment [30,31]. It should also be noted that all hydrofluoric acid procedures were conducted under certified chemical hood conditions, followed by immediate neutralization and disposal according to institutional hazardous waste protocols.

Studies on resin nanoceramics and polymer-infiltrated ceramic networks (PICNs) similarly show that higher HF concentrations create deeper etching patterns and greater microretentive potential compared with lower concentrations [22,23,27]. Tokgöz Çetindağ et al. [28] and Niizuma et al. [7] demonstrated that HF concentrations above 4.5% generate more distinct microcavities and topographical heterogeneity, supporting our ESEM observations in Groups 4 and 5. Therefore, the micromorphological features observed in the present study, although interpreted qualitatively, are compatible with previous reports on HF-induced structural changes in hybrid CAD/CAM materials.

EDS analysis showed increases in fluorine content and reductions in silicon and oxygen following HF conditioning, particularly in the 9.5% HF groups. Similar elemental trends were reported by Miranda et al. [2], who observed that HF etching of hybrid ceramics reduces silicon content while increasing fluorine uptake due to the formation of fluoride-containing reaction products. However, the degree of elemental change is strongly influenced by HF concentration, exposure time, and the intrinsic composition of the material [7,27].

An interesting pattern emerged in the current study: Group 4 (9.5% HF, 20 s) presented higher fluorine levels than Group 5 (9.5% HF, 60 s), suggesting potential over-etching effects. Comparable findings were described by Eldafrawy et al. [3] and Lise et al. [9], who noted that prolonged HF exposure can disrupt the newly formed fluoride-rich superficial layer, leading to rinsing-induced loss of reaction products. Pucci et al. [27] also highlighted that longer etching does not always correlate with increased chemical reactivity and may even lead to weakened surface integrity. These parallels reinforce the plausibility of the trends identified in our semi-quantitative EDS results. Other investigations confirm that over-etching may compromise surface stability by removing newly formed silica-fluoride complexes [32].

Although the SBS values obtained in pilot this work must be interpreted strictly as descriptive due to the extremely limited sample size, the directional trends observed are consistent with established principles. Groups treated with 9.5% HF generally presented higher SBS values than sandblasting alone, in line with results reported by Barutcigil et al. [6] and Fathy et al. [22], who showed that combining mechanical and chemical conditioning enhances resin cement interlocking and improves immediate bond strength. Comparable increases in repair bond strength after dual conditioning have been reported for multiple resin-matrix ceramics and nanoceramics [33].

Moreover, studies by Tokgöz Çetindağ et al. [28], Sismanoglu et al. [10], and Arkoy & Ulusoy [34] confirm that hybrid ceramics respond favourably to dual-treatment protocols, particularly when high filler content allows HF to selectively dissolve certain phases and facilitate functional monomer interaction. Our results follow the same directional tendencies, even though they cannot be statistically evaluated. Recent evaluations demonstrate that differences in resin content, filler distribution, and polymer architecture yield distinct bonding responses across hybrid CAD/CAM materials [10,35].

However, the variability in SBS values and the absence of a linear correlation between HF etching time and adhesion are consistent with findings by Niizuma et al. [7], who observed that HF-induced surface degradation does not always produce proportional gains in bond strength. In hybrid materials with complex microstructures, over-etching may weaken the surface and compromise the resin–substrate interface [23,36]. This aligns with the behaviour observed between Groups 4 and 5 in the present pilot study.

The improvements in bonding performance observed after combined mechanical and chemical conditioning can be attributed to synergistic surface effects. Sandblasting increases micromechanical retention through localized plastic deformation and particle-induced microcratering, while acid etching selectively dissolves exposed filler–matrix interfaces, enhancing surface energy and wetting. This combination improves adhesive infiltration and increases the effectiveness of silane chemistry in hybrid materials.

Therefore, relying entirely on manufacturer recommended protocols may result in insufficient bonding, especially in posterior applications where mechanical problems including debonding, marginal leakage, or restorative fracture are more common [16,18].

Complex occlusal dynamics, heat cycling, and constant masticatory stresses all apply to the posterior area. Bonding techniques must guarantee a solid adhesive contact able of resisting functional degradation over time under these conditions. The increased shear bond strength gained with the dual-treatment approach suggests the clinical need of modifying material preparation methods, especially as recent studies confirm that different surface treatments affect repair strength variably depending on the hybrid material used [32,34].

In addition, the topographic changes that were observed in the etched samples indicate that the resin penetration and hybrid layer formation have been enhanced. These mechanisms have been previously associated with stronger adhesive interfaces and reduced failure rates as further demonstrated by Degirmenci et al., where surface modification techniques improved mechanical integration [37].

Although the present results support the superior bonding results achieved by combining airborne-particle abrasion with hydrofluoric acid (HF) etching for Grandio Discs, this result should be interpreted considering the material’s specific composition and microstructure. Highly filled hybrid nanoceramic Grandio Discs have ~86% inorganic filler [38] content by weight, which increases surface microroughness following mechanical and chemical conditioning.

Other hybrid ceramics and CAD/CAM composite materials, such VarseoSmile Crown Plus (BEGO GmbH & Co. KG, Bremen, Germany), a 3D-printed hybrid crown material, or Vita Enamic (Vita Zahnfabrik H. Rauter GmbH & Co. KG, Bad Säckingen, Germany), a polymer-infiltrated ceramic network (PICN), show notable structural variations that might affect their reaction to surface treatments. For instance, while the resin-rich structure of VarseoSmile might not provide the same degree of micromechanical retention after etching as Grandio, Vita Enamic’s interpenetrating ceramic–polymer network is less reactive to HF etching.

While airborne-particle abrasion improved bond strength along many hybrid materials, HF etching did not provide additional benefit for 3D-printed hybrid materials or PICNs and may even weaken some materials due to superficial degradation [36].

Similarly, Grangeiro et al. (2021) [29] examined the impact of various surface treatments on the outer layer of hybrid ceramics and determined that not all etching protocols uniformly boost adhesion. This suggests that chemical reactivity varies among materials and layers.

Furthermore, surface conditioning techniques significantly influenced the efficacy of repair bond strength on hybrid ceramics by microtensile testing; HF etching provided little benefit unless the filler exposure of the material was optimized by previous air particle abrasion [39].

Moreover, Martinez et al. emphasized that surface preparation is largely responsible for the success of luting techniques using self-adhesive resin cements for 3D-printed hybrid restorations; however, overly aggressive etching could compromise surface integrity and reduce the long-term stability of the restoration [40].

The new generation of hybrid ceramic materials are joining the advantages of mechanical strength [40] and good biocompatibility [41,42,43,44,45]. Taken together, the results of our research show that although useful for Grandio, the present bonding technique cannot be used generally to all hybrid CAD/CAM materials. Selection of surface treatment techniques depends on careful evaluation of the material-specific microstructure and composition to guarantee accurate and strong adhesion.

This article offers important new perspectives on the influence of different surface treatments on the bond strength of CAD/CAM hybrid nanoceramics, but however some important limitations and methodological considerations should be discussed.

The present investigation was intentionally designed as a pilot feasibility study, with the primary objective of exploring whether combined mechanical and chemical surface conditioning protocols cause measurable micromorphological and elemental changes in a highly filled CAD/CAM hybrid nanoceramic, and whether these changes are linked to observable trends in adhesive behaviour. Accordingly, the study was not meant to provide statistically validated bond strength values or to establish clinically generalizable thresholds.

Within this context, shear bond strength (SBS) testing was used as a supplementary exploratory tool rather than as a primary mechanical endpoint. The SBS measurements were used to qualitatively determine if the surface modifications observed through ESEM and EDS showed any related trends in adhesion. This method was chosen to support a comprehensive interpretation of surface chemistry, micromorphology, and initial adhesive performance, rather than to draw definitive mechanical conclusions.

A significant limitation of the current study is the restricted number of specimens in each experimental group, which prevents inferential statistical analysis and restricts the broad applicability of the SBS values. Using two CAD/CAM discs per protocol, each bonded to two enamel surfaces, was chosen to reduce biological variability and enhance methodological consistency within the constraints of a pilot design. Nevertheless, the dataset generated should be viewed strictly as descriptive, and any observed differences between conditioning protocols should be considered as suggestive trends rather than statistically proven effects.

The allocation of both buccal and lingual enamel surfaces from the same tooth was used to reduce variability and to maintain comparable enamel prism orientation. While this approach works for preliminary feasibility testing, it is acknowledged that future studies aimed at statistical validation should include a larger number of independent specimens, avoid surface-sharing methods, and incorporate appropriate sample size calculations.

Despite these limitations, the current pilot study offers valuable methodological insights by identifying surface conditioning parameters that cause distinct micromorphological and chemical changes, and by showing that these alterations are linked to observable trends in adhesive performance. The results help guide the design of future large-scale studies, where optimized conditioning protocols, extended ageing procedures, and statistically powered experimental designs can be used to confirm the clinical relevance of the observed effects. Several of these aspects are further detailed in the dedicated “Limits of the Study” section.

### Limits of the Study

While this research provides relevant insights into the influence of surface conditioning protocols on a specific hybrid nanoceramic, several limitations must be acknowledged:

Firstly, all shear bond strength tests were carried out in vitro under controlled laboratory settings using extracted human molars. Although this method guarantees repeatability, it cannot completely reproduce the complex oral environment in which elements such as moisture, heat cycling, mechanical fatigue, and long-term ageing can affect adhesive performance.

Secondly, just one kind of CAD/CAM hybrid nanoceramics—Grandio disc—was tested, so the material-specific reaction to surface treatments could restrict the generalizability of our results. Different compositions and filler distributions of other hybrid ceramics or nanoceramic composites would affect their response to the same treatment techniques. Comparisons between subtractively and additively manufactured resin-matrix ceramics confirm substantial material-dependent variation in surface reactivity and adhesive behaviour [36,46]. A relatively small number of specimens per group (*n* = 2) was used for surface characterization, which limits the statistical power and generalization of the results. The present study was designed as a preliminary proof-of-concept investigation aimed at observing micromorphological and elemental trends rather than establishing statistically validated differences. Because of the limited sample size, no inferential statistical tests (e.g., ANOVA, *t*-tests) were applied. Descriptive parameters (individual values, mean and standard deviation) were reported to illustrate observable trends rather than statistically validated differences. Therefore, the outcomes should be interpreted as qualitative indications of the influence of surface conditioning on hybrid nanoceramic adhesion. Future studies with larger sample sizes will address this limitation.

Thirdly, the present study did not evaluate the long-term stability of the adhesive interface under conditions of either thermocycling or extensive water storage. Since thermomechanical and pH-cycling ageing can profoundly influence bonding durability in hybrid nanoceramics, incorporating such protocols is essential for future research [47].

At last, the EDS and ESEM studies were mostly applied for semi-quantitative surface characterization with qualitative aspects. Although informative, more sophisticated methods (such as atomic force microscopy or nano-indentation) might clarify the link between surface topography and micromechanical retention. Laser-assisted conditioning has also shown variable enhancement of resin–ceramic adhesion, further underscoring the complexity of optimizing surface treatment [48]. Further quantitative analyses, including 3D profilometry and micro-CT evaluation, would be useful to better correlate surface morphology with adhesive performance. These techniques will be considered in future investigations to confirm the microstructural effects observed in the present study. EDS results for oxygen and carbon are semi-quantitative due to the low excitation efficiency of light elements and the compositional heterogeneity of CAD/CAM hybrid nanoceramics, which contain both polymeric and glass-ceramic phases.

Future research addressing these limitations is needed to validate and expand upon the findings presented.

In summary, the improved micromechanical retention, chemical reactivity, and increased SBS values observed following dual treatment highlight the necessity of material-specific and clinically contextualized adhesive protocols in CAD/CAM restorative dentistry.

## 5. Conclusions

Within the limitations of this pilot feasibility study, several qualitative observations can be drawn regarding the influence of surface conditioning protocols on CAD/CAM hybrid nanoceramics. The combination of hydrofluoric acid (HF) etching with airborne-particle abrasion resulted in descriptively higher shear bond strength (SBS) values compared to sandblasting alone.

Micromorphological evaluation via ESEM revealed that increasing HF concentration and exposure time led to progressive surface roughening and porosity, facilitating improved micromechanical interlocking. Corresponding EDS analyses confirmed a significant uptake of fluorine and modification of silicon and oxygen levels in groups treated with 9.5% HF, indicating more pronounced chemical reactivity at the interface. By contrast, low-concentration HF (4.5%) produced limited surface changes, suggesting suboptimal conditioning when used alone or for shorter durations.

Shear bond strength testing confirmed that dual conditioning protocols, especially with 9.5% HF for 20–60 s, enhanced adhesion, yielding SBS values between 5.01 and 6.14 MPa. In comparison, sandblasting-only samples showed significantly lower bond strengths (~1.8 MPa), suggesting that mechanical treatment alone may not ensure reliable adhesion in clinical applications involving occlusal stress.

Notably, the data also reflect a nonlinear relationship between HF exposure time and bond strength: over-etching (e.g., 60 s with 9.5% HF) did not necessarily yield the highest SBS, highlighting the importance of optimizing treatment parameters rather than assuming “more is better.” The lack of a linear relationship between HF exposure time and SBS, and the possible signs of over-etching at prolonged exposure times, underline the need for carefully optimized conditioning parameters that balance reactivity with structural preservation.

From a clinical perspective, these results suggest that optimized dual-treatment protocols may extend the longevity and reliability of CAD/CAM restorations, especially when predictable bonding is essential. Future investigations should incorporate thermocycling, long-term ageing, and broader material comparisons. Such studies are necessary to validate these preliminary trends and determine whether the observed surface effects translate into consistent improvements in adhesive performance.

The conclusions of this study apply specifically to Grandio disc hybrid nanoceramics and should not be extrapolated to all CAD/CAM hybrid materials, which may respond differently due to compositional and microstructural variability. Nevertheless, the outcomes illustrate the importance of material-specific surface conditioning protocols and offer a basis for designing larger, statistically powered investigations aimed at establishing reliable and reproducible adhesive strategies for hybrid CAD/CAM restorations.

Recent literature confirms that CAD/CAM hybrids with differing filler architectures exhibit distinct responses to etching parameters and require individualized conditioning regimens [15,19].

Key conclusions are as follows:

- Surface topography and elemental composition are critical factors in achieving durable adhesive interfaces in hybrid CAD/CAM materials.
- Dual conditioning with sandblasting and HF etching produces synergistic effects, enhancing both micromechanical and chemical retention.
- Manufacturer-recommended minimalistic approaches relying solely on air abrasion may be insufficient, particularly for posterior restorations or high-load areas.
- The adhesion side of CAD/CAM processed dental restorations made from hybrid nanoceramics should also be conditioned by acid etching to enhance the bonding to hard dental tissues.
- Over etching the luting surface of CAD/CAM hybrid nanoceramics restorations (by the mean of concentration or time of exposure to hydrofluoric acid) decrease the overall resistance of the joint.
- Adhesive strategies must be tailored to the specific composition and microstructure of the restorative material—findings from Grandio disc cannot be extrapolated to all hybrid ceramics.

## Figures and Tables

**Table 1:** Surface treatment protocols applied to each sample group.

	Surface Preparation		
Sample	Sandblasting	HF	Time
Group 1	30 µm Al_2_O_3_	-	-
Group 2	30 µm Al_2_O_3_	4.5%	20 s
Group 3	30 µm Al_2_O_3_	4.5%	60 s
Group 4	30 µm Al_2_O_3_	9.5%	20 s
Group 5	30 µm Al_2_O_3_	9.5%	60 s

**Table 2:** SBS values.

Sample	Side	KgF	N	MPa
1	side 1	5.34	52.36	1.85
1	side 2	5.43	53.24	1.88
2	side 1	16.37	160.55	5.68
2	side 2	17.69	173.56	6.14
3	side 1	13.36	131.07	4.64
3	side 2	11.62	114.0	4.03
4	side 1	14.44	141.68	5.01
4	side 2	15.38	150.86	5.34
5	side 1	9.75	95.67	3.38
5	side 2	8.8	86.37	3.06

## Data Availability

The original contributions presented in the study are included in the article. Further inquiries can be directed to the corresponding authors.

## Abbreviations

The following abbreviations are used in this manuscript:

CAD/CAM	Computer-Aided Design/Computer-Assisted Manufacturing
HF	Hydrofluoric Acid
APA	Airborne-Particle Abrasion
SBS	Shear Bond Strength
ESEM	Environmental Scanning Electron Microscopy
EDS	Energy-Dispersive X-ray Spectroscopy
